# How Ubiquitin Unfolds after Transfer into the Gas Phase

**DOI:** 10.1007/s13361-012-0370-6

**Published:** 2012-04-03

**Authors:** Owen S. Skinner, Fred W. McLafferty, Kathrin Breuker

**Affiliations:** 1 Department of Chemistry and Chemical Biology, Cornell University, Ithaca, NY 14853-1301 USA; 2 Institute of Organic Chemistry and Center for Molecular Biosciences Innsbruck, University of Innsbruck, Innrain 80/82, 6020 Innsbruck, Austria

**Keywords:** Electron capture dissociation, Protein, Ubiquitin, Gas phase structure

## Abstract

The structural evolution of ubiquitin after transfer into the gas phase was studied by electron capture dissociation. Site-specific fragment yields show that ubiquitin’s solution fold is overall unstable in the gas phase, but unfolding caused by loss of solvent is slowest in regions stabilized by salt bridges.

## Introduction

Electrospray ionization (ESI) has revolutionized mass spectrometry (MS) by extending its applicability to large nonvolatile molecules. Although covalent bonding and, thus, molecular weight information, is usually preserved in ESI, retention of the noncovalent bonding of the solution structure has been demonstrated far less frequently [1–3]. ESI/MS is widely used to screen for ligands that bind to target proteins [4]; a better understanding of the effect of ESI on the noncovalent bonding in protein–ligand complexes would help evaluate the reliability of screening results. Unlike collisionally activated dissociation during which different conformers of the same net charge can rearrange to structurally similar transition states prior to fragmentation [5], electron capture dissociation (ECD) [6] can provide site-specific information on the structure and stability of gaseous protein ions [7]; key to this is the retention of noncovalent bonds during ECD [2, 3, 7–10]. ECD and “native ECD” [11] experiments showed that solution structure is not necessarily preserved in the gas phase; the highly stable protein cytochrome *c* (ΔG for global unfolding in solution = 42 ± 2 kJ/mol at 30 °C) [12] unfolds on a milliseconds timescale after transfer into the gas phase [13], whereas the fold of the moderately stable protein KIX (ΔG = 12 ± 1 kJ/mol at ∼27 °C) [14] is preserved in the gas phase on a timescale of at least 4 s [2]. Unfolding of cytochrome *c* after desolvation was also observed in ion mobility spectrometry (IMS) studies [15]. The 7+ ions of ubiquitin, whose structure is believed to most closely resemble its native fold, have been studied extensively [16]. In IMS experiments, Clemmer and coworkers observed substantial unfolding of 7+ ubiquitin ions on a timescale of 0.1 s after ESI [17], whereas a recent study by Wyttenbach and Bowers reports conditions under which these ions do not unfold in 0.1 s [18]. A previous ECD study has provided site-specific data on the gas phase structure and folding/unfolding of the 7+ ubiquitin ions [7], but these were probed >40 s after desolvation and were shown to be stable gas phase structures substantially different from those in solution. Here we report ECD data that reflect the initial structural changes of ubiquitin 7+ ions after transfer into the gas phase.

## Experimental

Experiments were performed on a 7 T Fourier transform-ion cyclotron resonance (FT-ICR) mass spectrometer (Bruker, Vienna, Austria). After ESI (1.5 μL/min) of a 2 μM ubiquitin (Sigma-Aldrich, Vienna, Austria) solution in 79:20:1 H_2_O/CH_3_OH/CH_3_COOH at pH 3 (in which ubiquitin retains its native fold [19]) under conditions to minimize ion activation by collisions with background gas, (M + 7H)^7+^ ions were accumulated in the first hexapole (0.1 s), selected by *m*/*z* in the quadrupole, accumulated in the second hexapole (0.1 s), trapped in the FT-ICR cell, isolated, and irradiated with electrons (0.8 eV) for 30 ms. This experiment was repeated with 5 s ion storage in the FT-ICR cell prior to ECD, and with collisional activation at the head of the second hexapole. 500 scans were added for each spectrum. Data analysis utilized SNAP2 (Bruker, Vienna, Austria) and mMass [20] software. Site-specific fragment yields were calculated as %-values relative to all ECD products, considering that backbone cleavage gives a pair of complementary ***c*** and ***z*** ^•^ ions (***a*** ^•^, ***y*** ions were also included for normalization but not shown in the figures because of their marginal abundance totaling to <4 %): 100 % = 0.5·[***c***] + 0.5·[***z*** ^•^] + 0.5·[***a*** ^•^] + 0.5·[***y***] + [reduced molecular ions and loss of small neutral species from the latter].

## Results and Discussion

**Figure 1.**
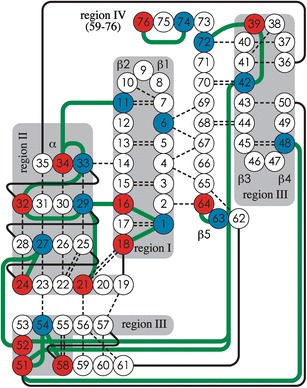
Intramolecular electrostatic interactions of Ubiquitin (PDB entry 1D3Z) with regions I (residues 1–18), II (19–35), III (36–58), and IV (59-76); basic (K, R, N-terminus) and acidic (D, E, C-terminus) residues in blue and red, respectively; hydrogen bonds: dashed lines, solid black lines connect sequence neighbors, green lines: possible salt bridges

**Figure 2.**
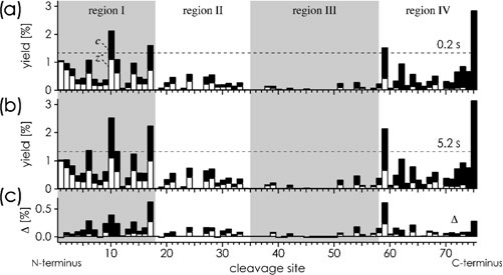
Site-specific yields **(a)**, **(b)** of ***c***, ***z*** ^•^ fragments from ECD of ubiquitin (M + 7H)^7+^ ions 0.2 s **(a)** and 5 s **(b)** after transfer into the gas phase and yield difference **(c)**; dashed horizontal lines illustrate calculated yields for random fragmentation

In ubiquitin’s native structure, only residues 72–76 are not involved in noncovalent bonding (Figure 1). If this fold were preserved in the gas phase, only fragments from cleavage at sites 72–75 would be found in the ECD spectra because intramolecular noncovalent bonding would prevent separation of all other fragments [2, 7]. However, ECD of the 7+ ions shows abundant ***c***, ***z*** ^•^ fragment ions from cleavage at many other sites (Figure 2), indicating substantial unfolding after desolvation. Specifically, region I (residues 1–18) that includes β-strands 1 and 2 and region IV (residues 59–76) that includes β-strand 5 have unfolded in ∼57 % and ∼47 % of the ions after 0.2 s, respectively (Figure 2a). The extent of unfolding was calculated from integrated ***c***, ***z*** ^•^ fragment ion yields in each region versus those expected from random cleavage in a fully extended structure (100 %/75 = 1.33 % yield per cleavage site). The similarity of the values for regions I and IV suggests that they have separated from the hydrophobic core in the same unfolding event. From the data in Figure 2a, regions II and III have unfolded in just 17 % and 5 % of the 7+ ions within 0.2 s, respectively.

The site-specific differences of fragment yields (Figure 2c) from ECD 0.2 and 5.2 s after transfer into the gas phase show a pattern very similar to that from ECD after 0.2 s (Figure 2a), indicating that the initial unfolding during the first 0.2 s continues during the next 5 s in the same manner. Exceptions to this are the fragment yields at sites 1–5, which do not increase in proportion from 0.2 to 5.2 s (Figure 2c), indicating that unfolding of residues 1–5 is largely complete after 0.2 s. After 5.2 s, 71 %, 22 %, 7 %, and 57 % of the ions have unfolded in regions I–IV, respectively, suggesting a regional order of stability of III > II > IV > I.

**Figure 3.**
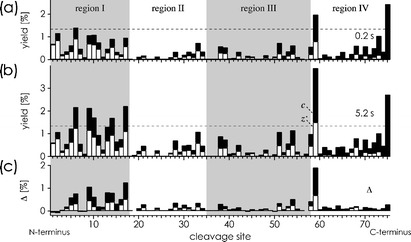
As in Figure 2, but with collisional activation after 0.1 s (105 eV laboratory frame energy)

Collisional activation of the 7+ ions 0.1 s after ESI under otherwise identical experimental conditions gave the fragmentation patterns shown in Figure 3a, b. The data indicate an extent of unfolding of 49 %, 13 %, 17 %, and 41 % in regions I–IV after 0.2 s, respectively. Again, the fragment yield differences from ECD 0.2 and 5.2 s after transfer into the gas phase (Figure 3c) show a pattern very similar to that from ECD after 0.2 s, indicating that the unfolding during the first 0.2 s continues during the next 5 s, with 81 %, 22 %, 27 %, and 58 % of the ions unfolded in regions I–IV after 5.2 s, respectively.

Apparently, the 26 hydrogen bonds of ubiquitin’s β-sheet (Figure 1) are insufficient to preserve the solution fold after desolvation: regions I and IV unfold in about half of the 7+ ions after just 0.2 s. On the other hand, region III, which is the most heterogeneous in terms of secondary structure, is unfolded in only 5 % and 7 % of the ions after 0.2 and 5.2 s, respectively. Substantial collisional activation is required to increase these fractions to 17 % and 27 %, indicating extensive stabilization. Region II that comprises the α-helix is unfolded in only ∼20 % of the ions at 0.2 and 5.2 s without collisional activation, suggesting that ubiquitin’s α-helix structure is more stable than its β-sheet in the absence of solvent. However, a recent study of KIX showed that helix stability in the gas phase critically depends on additional stabilization by salt bridges [2]. Salt bridges as stabilizing structural motifs have also been suggested by Williams and coworkers for gaseous peptide ions [21].

The very same collisional activation that increases the fraction of 7+ ions unfolded in regions I and III by 10 % and 20 % after 5.2 s, respectively, does not further unfold regions II (22 % at 0.2 and 5.2 s) and IV (57 % at 0.2, 58 % at 5.2 s). For region II, this is consistent with additional stabilization of the α-helix by salt bridges, but structural changes are nevertheless apparent from the different ECD fragmentation patterns for region II at 0 and 105 eV (Figure 2a, b and Figure 3a, b). These possibly involve rearrangements of salt bridges upon activation while retaining the helical structure. Similar rearrangements are indicated for the far less compact region IV, but not for regions I and III, whose ECD fragmentation patterns at 0 and 105 eV are very similar (Figure 2a, b and Figure 3a, b).

Salt bridges, formed during the electrospray process or already present in solution, appear to play a dominant role in stabilizing regions of the native fold of ubiquitin after transfer into the gas phase. Six salt bridges are possible in the most stable region III that comprises 23 residues. The second most stable region II (17 residues) has five possible salt bridges. Only two salt bridges are possible in the least stable regions I and IV, respectively. Specifically, the N- and C-terminal β-strands 1 and 5 are held together by hydrogen bonds, but no salt bridges can provide additional stabilization.

Regions I and IV comprise 36 out of ubiquitin’s 76 residues; their unfolding in just 0.2 s should result in a substantial increase in collision cross section for about half of the 7+ ions, consistent with IMS data that indicate unfolding of “compact” ions (∼1000 Å^2^) to form ∼50 % “partially folded” structures with cross sections between 1120 and 1500 Å^2^ 0.2 s after ESI [17]. However, this study also finds ∼15 % “elongated” structures (>1500 Å^2^) after 0.2 s, increasing to ∼25 % in 5 s. The proportion of elongated structures indicated by our ECD data is smaller, with only 7 % of the 7+ ions unfolded in region III after 5 s. The ESI and ion transmission conditions of these IMS experiments appear to result in more extensive unfolding than those used here. Although the timescale of unfolding of ubiquitin after transfer into the gas phase evidently depends on experimental conditions [17, 18], the order of unfolding established here (region I > IV > II > III) is dictated by the extent to which the loss of hydrophobic bonding is compensated by electrostatic interactions, in particular salt bridges, in different regions of the native fold. Stabilization by salt bridges is also indicated for the 7+ ions probed 40 s after desolvation, which gave few fragments from cleavage at sites 11–58 [7], consistent with disintegration of the native fold and folding into stable gas phase structures [15, 17, 22].

The ECD data presented here complement IMS data, and allow for a structural interpretation of the unfolding of ubiquitin 7+ ions after transfer into the gas phase. Solvent removal destabilizes the native fold, with regions I and IV unfolding markedly faster than regions II and III. We attribute the observed regional stabilization in large part to salt bridges, consistent with recent findings for the three-helix bundle protein KIX [2]. The extent to which electrostatic interactions and, in particular, salt bridges can compensate for the loss of hydrophobic bonding largely defines the timescale available for structural probing of a solution fold in the gas phase.
